# The moderating effect of gender on the relationships between obesity, well-being, and stress perception in Korean adolescents

**DOI:** 10.1186/s12889-021-11894-3

**Published:** 2021-10-14

**Authors:** Nam Su-Jung, Park Jong-Ho

**Affiliations:** 1 Department of Home Economics Education, College of Education, Jeonju University, Jeonju, Republic of Korea; 2 365MC Obesity Clinic, Soowon, Republic of Korea

**Keywords:** Adolescents, Obesity based on BMI, Obesity based on perception, Gender, Well-being, Stress perception

## Abstract

**Background:**

Children and adolescents with obesity are more likely to become adults with obesity. Therefore, obesity prevention in adolescence is essential for eliminating complications associated with obesity, which can affect health throughout the lifespan. This study examined the influences of adolescents’ obesity based on BMI and that of obesity based on adolescents’ perception of their well-being and stress, as well as the moderating effect of gender on these influences.

**Methods:**

Data were analyzed for 61,861 adolescents aged 12–18, who participated in the 2019 Korean Adolescent Health Behavior Survey, an online self-administered questionnaire. Adolescents’ obesity based on perception was based on their self-rating as underweight, healthy weight, or obese. Chi-squared tests were used to determine whether well-being and stress perception were related to obesity based on BMI and obesity based on perception, and a general linear model was used to examine the main and interaction effects of obesity based on BMI, obesity based on perception, and gender on well-being and stress perception.

**Results:**

Obesity based on BMI and obesity based on perception coincided in 58.7% of the sample. However, the degree of obesity was overestimated and underestimated by 19.2 and 24.3% of the sample, respectively. Obesity based on BMI and obesity based on perception varied by gender, age, economic status, and academic achievement. The main effect of obesity based on BMI was not statistically significant on both well-being and stress perception, and only the main effect of obesity based on perception was statistically significant on stress perception. The interaction between obesity based on perception and gender was significant for well-being and stress perception. Herein, males scored higher on well-being and lower on stress perception. However, the association patterns were similar for males and females, with significant differences between underweight, healthy weight, and overweight/obese; however, for only females, there was no difference in well-being scores between underweight and those who perceived themselves as having a healthy weight.

**Conclusion:**

The well-being and stress perception are influenced by obesity based on perception rather than obesity based on BMI, and this influence varies according to gender in adolescents.

## Background

Childhood obesity has been identified as one of the major public health problems in the United States and many industrialized countries of the world [1, 2]. In Korea, the obesity rate among children and adolescents is increasing every year. As of 2018, the obesity rate of 13–18 years old was 14.4% [3]. The association of obesity with morbid outcomes makes it a public health concern for children and adolescents [4]. In addition, research indicates that children with obesity are much more likely to suffer from cardiovascular and digestive diseases in adulthood [5]. An increase in body fat also exposes an adolescent to an increased risk of numerous forms of cancer, such as breast, colon, esophageal, kidney, and pancreatic cancers [6]. Therefore, obesity prevention in adolescence is essential for eliminating the complications associated with obesity that can affect health throughout the lifespan [7].

Furthermore, studies have demonstrated that, in addition to its impact on physical health, obesity increases the risk of mental illnesses such as depression [8], with a direct relationship having been found between the two [9]. These studies, which focused on the relationship between obesity and health, suggest that, as the level of obesity increases, physical and mental health decline, and as the degree of dissatisfaction with one’s body type increases, the quality of life decreases [10] and disease morbidity increases [7]. Numerous studies have analyzed the relationship between obesity and the prevalence of morbidity, mortality, and quality of life; however, most of these studies have focused on obesity based on the body mass index (BMI), (OBB), and not on obesity based on perception (OBP). OBP refers to the perception of one’s body type as underweight, healthy weight, overweight, or obese, regardless of one’s BMI. Empirical studies have suggested that there is a discrepancy between OBB and OBP [11, 12]. One previous study found a considerable discrepancy between OBB and OBP, with women more likely than men. and younger adults more likely than middle-aged people to overestimate their OBB [13].

Overall, however, studies on the relationship between obesity and psychological problems have shown contradictory results. Some studies have suggested that if adolescents perceived themselves as obese, then their levels of anxiety and stress increase, their level of depression increases, and they have lower self-esteem [14, 15]. In one study, adolescents with obesity based on BMI were more likely to have psychological problems [16], but another study found no significant relationship between the likelihood of psychological problems and OBB [17]. To clarify the relationship between obesity and psychological problems, recent studies have utilized measures of OBP rather than OBB [18].

Adolescents are interested in body shape and appearance and compare their body shape to that of their peers, which suggests that obesity in adolescence not only is a health problem but may also negatively affect emotional development. In adolescence, a distorted self-perception of body shape is a risk factor for psychological problems and inappropriate methods of weight control [19]. In particular, excessive weight control and poor eating habits due to distorted body perception and body dissatisfaction have been found in girls [20]. Overall, girls were found to be less satisfied with their weight and body shape than boys [21]. Previous studies on Chinese [22], American [23], and European [24] women reported that they overestimated their weight by 21.02, 23.50, and 23.80%, respectively; in all cases, the proportion of women overestimating their weight was higher than that of men. Although their BMIs were appropriate, the women reported utilizing various methods to lose weight [25]. This finding suggests that women are more affected by the perception of being thin, consistent with its emphasis in the media as the ideal body image [26]. Distorted body perception is related to body dissatisfaction and attempting unhealthy weight control strategies, which lead to body dysmorphic disorder, health-obsessive behaviors, low quality of life, and eating disorders [27–29]. Also, distorted body perception and weight control strategies are related to the prevalence of psychological problems, as people with inaccurate perceptions of their body shape who use medical procedures for weight control could be at a higher risk of psychological disorders [30].

The disparity between OBB and OBP varies by gender. For example, an empirical study reported that 38.3% of girls with healthy weight subjectively perceived themselves to be overweight, while 32.8% of overweight boys perceived themselves to be underweight or having a healthy weight [31]. The relationship between self-esteem and the disparity between OBB and OBP may also vary by gender. One study found that the more girls perceived themselves as underweight rather than having a healthy weight or obese, the higher their self-esteem; however, the more boys perceive themselves as underweight rather than obese, the lower their self-esteem [18]. Overall, an inaccurate or distorted perception of one’s body negatively affected both girls and boys.

Therefore, this study examines the effects of OBB and OBP on the well-being and stress perception of adolescents and whether the relationship between them is moderated by gender. To this end, the main and interaction effects of OBB, OBP, and gender are investigated. To clarify the relationship between obesity and psychological problems in adolescents, the study poses the following research questions: (1) What are the main effects of OBB and OBP on well-being and stress perception? (2) Is gender a moderator in the relation of OBB or OBP with well-being and stress perception?

## Methods

### Data

The current sample consisted of 61,861 Korean adolescents aged 12 to 18 years who participated in the 2019 Korean Adolescent Health Behavior Survey (KAHB), an annual survey conducted by the Korea Centers for Disease Control and Prevention. The KAHB is an anonymous and self-administered online questionnaire used to identify adolescents’ health behaviors, such as their physical activity and eating habits. All experimental protocols were approved by the Korea Centers for Disease Control and Prevention and all research methods were carried out in accordance with its guidelines and regulations. Additionally, w**ritten informed consent** was obtained from all respondents prior to the survey by the Korea Centers for Disease Control and Prevention.

A stratified cluster sample was extracted for the current study. Stratification variables were used for the regional and school levels (middle school, general high school, specialized high school). The distribution of the sample schools by region, city size, and school characteristics was determined by applying the proportional distribution method so that the population composition ratio and sample composition ratio for each stratification variable matched. Stratified cluster extraction was used, in which the primary extraction units were schools, and the secondary extraction units were classes.

### Measurement

The respondents were asked to indicate their gender as male or female and age in years. In addition, students’ economic status was measured by their responses to the question, “What is the economic status of your family?” and academic achievement was measured by their responses to the question, “How has your academic achievement been during the past year?” The response choices for both questions were lowest, lower-middle, middle, upper-middle, and highest.

The Centers for Disease Control and Prevention (CDC) recommends BMI based on age and sex as the best way to screen for childhood obesity [32]. In this study, according to CDC, adolescents were categorized as “obese” if they had a BMI ≥ 95th percentile, “overweight” if they had a BMI in 85th to <95th percentile, “healthy weight” if they had a BMI in the 5th to <85th percentile, and “underweight” if they had a BMI ≤ 5th percentile, based on the “2017 Growth Charts for Children and Adolescents,” which classifies BMI based on age and sex-specific cut-off values and is published by the Korea Disease Control and Prevention Agency and The Korean Pediatric Society [33, 34].

OBP was based on adolescents’ responses to the question of whether they viewed their body type as underweight, healthy weight, or obese.

Previous studies indicate that short scales of mental health are increasingly used in epidemiological studies [35] to reduce the burden on respondents and simplify management and translation. Therefore, well-being and stress perception were each measured as a single item. Well-being was measured by responses to the question, “What is your usual well-being level?” on a 5-point Likert scale that ranged from 1 (strongly disagree) to 5 (strongly agree), and stress perception was measured by responses to the question, “How much stress do you feel during normal times?” on a 5-point Likert scale that ranged from 1 (strongly disagree) to 5 (strongly agree).

### Statistical analysis

A chi-squared test was employed to test whether the respondent characteristics were related to OBB and OBP. The general linear model was used to examine the main effects of gender, economic status, academic achievement, OBB, and OBP, in addition to the interaction effects of OBB, OBP, and gender on well-being and stress perception. SPSS 21.0 (Chicago, IL) was used to conduct all analyses.

## Results

### Classification of obesity

The distributions of OBB and OBP are shown in Table 1. OBB and OBP coincided in 51.5% of respondents (underweight = 7.3%, healthy weight = 34.5%, obese = 9.7%). However, 29.4% of the sample overestimated their OBB, and the highest proportion of these respondents, 20.4% of the sample, had a healthy weight but perceived themselves as obese. Conversely, 19.0% underestimated their obesity, and the highest proportion of these respondents, 18.0% of the sample, had a healthy weight but perceived themselves as underweight.

**Table 1:** Classification of Obesity

		Obesity based on perception			*χ*^2^	*p*
		Underweight	Healthy weight	Obese		
Obesity based on BMI	Underweight	4425 (7.3%)	505 (0.8%)	81 (0.1%)	28,398.608	.000
	Healthy weight	10,851 (18.0%)	20,785 (34.5%)	12,274 (20.4%)		
	Overweight	20 (0.0%)	470 (0.8%)	4853 (8.1%)		
	Obese	21 (0.0%)	130 (0.2%)	5846 (9.7%)		

### Associations between demographic characteristics and OBB

The distributions of gender, age, economic status, and academic achievement by OBB are shown in Table 2, and all of the associations were statistically significant.

**Table 2:** Demographic Characteristics by Obesity Based on BMI

Characteristic	Underweight	Healthy weight/	Overweight	Obese	χ^2^
	n (%)	n (%)	n (%)	n (%)	
Gender					412.538^***^
Male	2754 (55.0)	21,409 (48.8)	2703 (506)	3722 (62.1)	
Female	2257 (45.0)	22,501 (51.2)	2640 (49.4)	2275 (37.9)	
Age					273.122^***^
12	357 (7.1)	3509 (8.0)	613 (11.5)	412 (6.9)	
13	909 (18.1)	7476 (17.0)	861 (16.1)	827 (13.8)	
14	933 (18.6)	7429 (16.9)	793 (14.8)	868 (14.5)	
15	879 (17.5)	7337 (16.7)	834 (15.6)	1017 (17.0)	
16	821 (16.4)	7583 (17.3)	825 (15.4)	1171 (19.5)	
17	794 (15.8)	7555 (17.2)	1018 (19.1)	1185 (19.8)	
18	318 (6.3)	3021 (6.9)	399 (7.5)	517 (8.6)	
Economic status					160.604^***^
Lowest	104 (2.1)	926 (2.1)	142 (2.7)	223 (3.7)	
Lower-middle	585 (11.7)	4833 (11.0)	688 (12.9)	886 (14.8)	
Middle	2262 (45.1)	20,494 (46.7)	2445 (45.8)	2659 (44.3)	
Upper-middle	1510 (30.1)	12,990 (29.6)	1536 (28.7)	1641 (27.4)	
Highest	550 (11.0)	4667 (10.6)	532 (10.0)	588 (9.8)	
Academic achievement					257.824
Lowest	529 (10.6)	3872 (8.8)	566 (10.6)	761 (12.7)	
Lower-middle	1074 (21.4)	9464 (21.6)	1273 (23.8)	1569 (26.2)	
Middle	1425 (28.4)	12,749 (26.6)	1517 (28.4)	1693 (28.2)	
Upper-middle	1250 (24.9)	11,693 (26.6)	1352 (25.3)	1328 (22.1)	
Highest	733 (14.6)	6132 (14.0)	635 (11.9_	646 (10.8	

*Notes*: ^***^p < .000

### Associations between demographic characteristics and OBP

The distributions in gender, age, economic status, and academic achievement by OBP are shown in Table 3, and all of the associations were statistically significant. A higher proportion of males than females perceived themselves as underweight, but a higher proportion of females than males perceived themselves as obese.

**Table 3:** Demographic Characteristics by Obesity Based on Perception

Characteristic	Underweight	Healthy weight	Obese	χ^2^
	n (%)	n (%)	n (%)	
Gender				2282.057^***^
Male	19,356 (62.4)	21,016 (47.6)	22,334 (46.1)	
Female	11,682 (37.6)	23,172 (52.4)	26,162 (53.9)	
Age				406.171^***^
12	2594 (8.4)	3840 (8.7)	3540 (7.3)	
13	5426 (17.5)	7750 (17.5)	7384 (15.2)	
14	5450 (17.6)	7562 (17.1)	7560 (15.6)	
15	5260 (16.9)	7190 (16.3)	8220 (16.9)	
16	5196 (16.7)	7474 (16.3)	8744 (18.0)	
17	5042 (16.2)	7472 (16.9)	9234 (19.0)	
18	2070 (6.7)	2900 (6.6)	3814 (7.9)	
Economic status				867.715^***^
Lowest	688 (2.2)	802 (1.8)	1532 (3.2)	
Lower-middle	3534 (11.4)	4168 (9.4)	6802 (14.0)	
Middle	13,774 (44.4)	20,900 (47.3)	22,314 (46.0)	
Upper-middle	9524 (30.7)	13,134 (29.7)	13,420 (27.7)	
Highest	3518 (11.3)	5184 (11.7)	4428 (9.1)	
Academic achievement				869.500^***^
Lowest	2970 (9.6)	3604 (8.2)	5508 (11.4)	
Lower-middle	6468 (20.8)	9138 (20.7)	11,952 (24.6)	
Middle	8372 (27.0)	13,514 (26.3)	13,614 (28.1)	
Upper-middle	8372 (27.0)	11,628 (296.3)	11,870 (24.5)	
Highest	4858 (15.6)	6304 (14.3)	5552 (11.4)	

*Notes*: ^***^p < .000

### Effects of OBB and OBP on well-being and stress perception

**Fig. 1:**
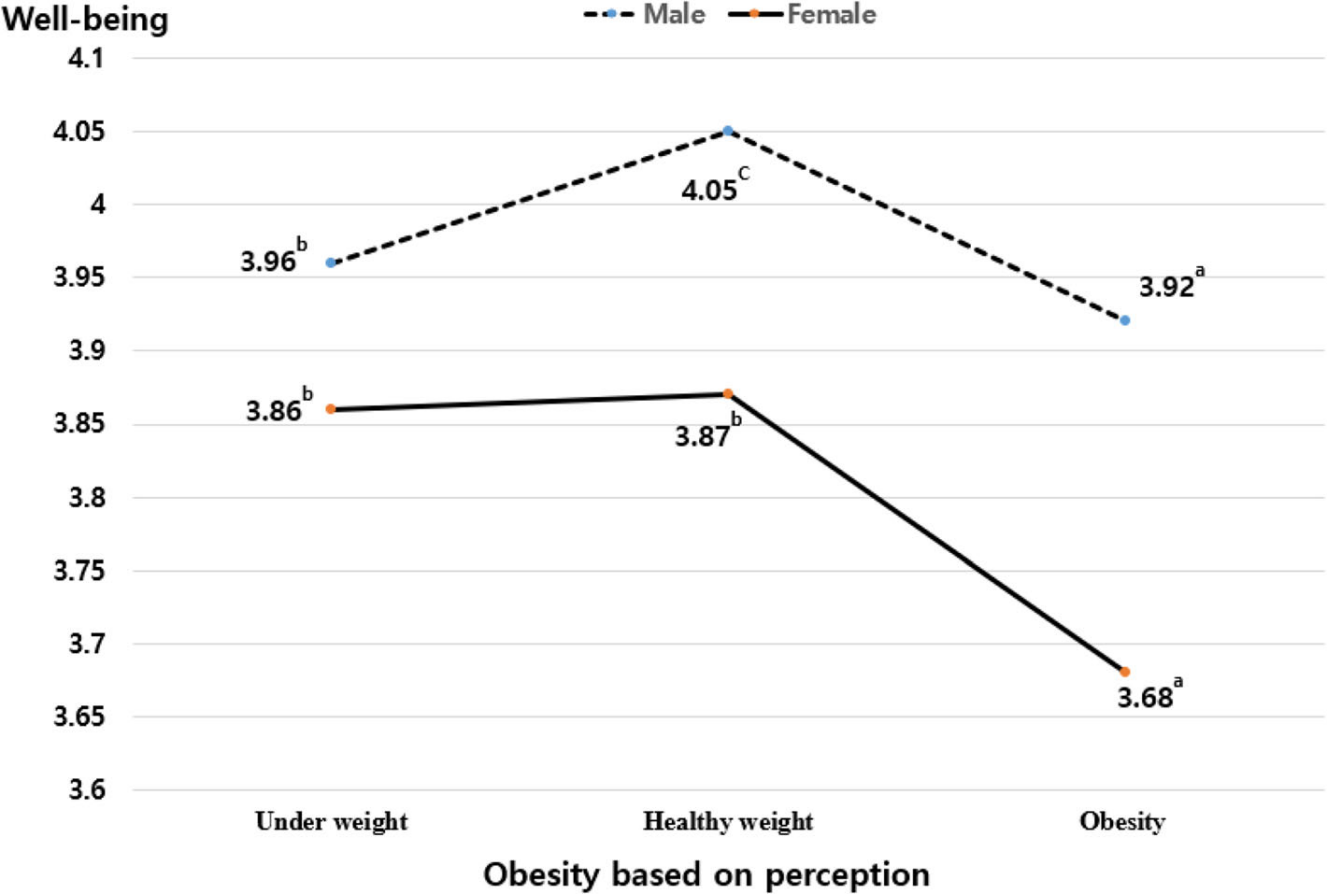
The effect of obesity based on perception and gender on well-being. The figure shows the interaction effect of obesity based on perception and gender on well-being among Korean adolescents. Note: ^a,b,c^In the Tukey test, the significance of the mean difference of each group was verified at the 0.05 level, and the degree of the mean difference for each group was expressed as a<b<c

**Fig. 2:**
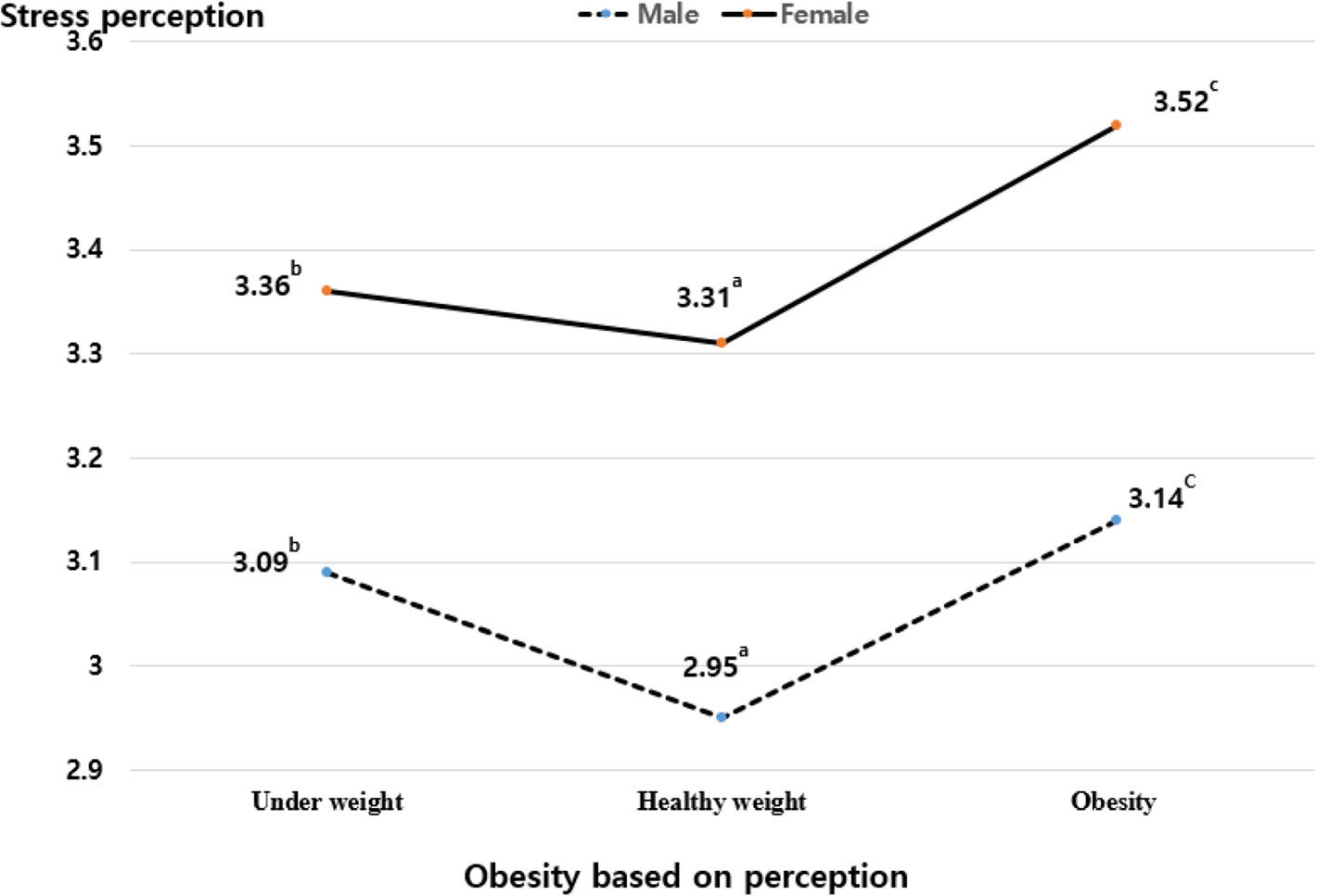
The effect of obesity based on perception and gender on stress perception. The figure shows the interaction effect of obesity based on perception and gender on stress perception among Korean adolescents. Note: ^a,b,c^In the Tukey test, the significance of the mean difference of each group was verified at the 0.05 level, and the degree of the mean difference for each group was expressed as a<b<c

The main effect of OBB was not statistically significant on both well-being and stress perception, and the main effect of OBP perception was statistically significant on them as shown in Table 4. The interaction effect of OBP and gender on well-being and stress perception was statistically significant (see Figs. 1 and 2); however, the interaction effect of OBB and gender on well-being and stress perception was not significant.

**Table 4:** Effects of Obesity based on BMI and Obesity based on perception on well-being and stress perception

	Well-being				Stress perception			
	SS	F	*p*	*η*_*p*_^*2*^	SS	F	*p*	*η*_*p*_^*2*^
Gender (A)	166.322	199.109	.000	.003	687.961	778.229	.000	.013
Age	114.646	137.027	.000	.013	75.703	85.637	.000	.008
Economic status: middle and higher	838.304	1003.557	.000	.016	458.101	518.209	.000	.009
1Academic achievement: middle and higher	536.591	642.367	.000	.011	175.763	198.825	.000	.003
Obesity based on BMI (B)	.464	.555	.645	.000	2.752	3.113	0.25	.000
Obesity based on perception (C)	4.499	5.386	.005	.000	7.375	8.343	.000	.000
A × B	1.365	1.634	.179	.000	1.046	1.184	.314	.000
A × C	5.353	6.408	.002	.002	6.006	6.794	.001	.003
B × C	.713	.854	.528	.000	2.400	2.714	.052	.000

Regarding well-being, males scored higher than females, and the scores of groups with perceptual obesity were the lowest. The difference between the means of each group was found to be statistically significant at the 0.05 level in the Tukey test. Among females, there was no difference in the well-being scores between respondents with perceptual underweight and perceptual healthy weight. Among those with perceptual obesity, well-being scores were significantly lower in females than in males. For stress perception, females scored higher than males. Moreover, both males and females with perceptual obesity had the highest stress perception scores, and the scores decreased in order for respondents with underweight and healthy weight.

## Discussion

Using the 2019 KAHB, this study examined the effects of OBB and OBP on well-being and stress perception and the moderating effect of gender on the relationship between them among Korean adolescents. In adolescence, the perception of one’s body type may be distorted due to the influence of mass media, and this distorted perception of body type may cause psychological problems and serve as a risk factor for inappropriate methods of weight control [19]. Previous research has suggested that a distorted perception of body type and indiscriminate weight control due to body dissatisfaction are more frequently found in females than in males [20], which is consistent with the results of this study. In this study, there was no significant difference in OBB between males and females. However, for OBP, the ratio of males in the group with underweight was higher than that for females, and in the group with obesity, the proportion of females was higher than that for males. These results are consistent with those of a previous study, in which researchers found that men were more likely than women to perceive their body shape correctly, with women tending to overestimate their OBB [36]. Similarly, in a study of middle and high school adolescents, gender was one of the strongest predictors of high school students’ perceptions of obesity [37]. Additional research has reported that female adolescents perceived their weight more negatively than male adolescents, were more interested in appearances than males, and perceived themselves as more obese than males [38, 39]. In addition, women were more likely to mistakenly perceive their bodies as obese because they compared themselves to the ideal thin body type delivered through mass media [40].

The results of the general linear model indicated that well-being and stress perception are influenced by OBP rather than OBB, and this influence varies according to gender. Well-being scores were the highest for both males and females with a healthy weight. For females, there was no significant difference in well-being between the group with underweight and the group with healthy weight; however, for males, the score of the group with underweight was lower than that of the group with healthy weight. Especially for females, the well-being scores for the group with obesity were significantly lower than for that the other groups. These results are consistent with those of a previous study, which reported that males who perceived themselves as underweight had low body satisfaction and self-esteem [18], and women who overestimated their weight reported the most negative psychological conditions including anxiety, self-esteem, body satisfaction, quality of life, and eating attitudes [30]. However, although the interaction effect between OBP and gender on stress perception was statistically significant, both men and women showed very similar results for stress perception; the scores were highest for the group with obesity and decreased in order for the groups with underweight and healthy weight.

In this study, the interaction effects of OBP and gender on well-being and stress perception were found to be statistically significant, but their effect sizes were small, at 0.02 and 0.03. In particular, because the results for stress perception were similar for women and men, it is difficult to completely exclude the possibility that the only valid main effects were those for OBP and gender. However, when interaction effects are present, it suggests that interpretation of the main effects is incomplete or misleading [41–43]. Therefore, it is necessary to consider an interaction effect, even if its effect size is small. In addition, many previous studies have reported a strong relationship between gender and body perception [19, 22, 23, 44, 45] and the importance of gender in relation to the mental health of people with a distorted perception of body weight [46, 47]. For example, one study found that overestimated OBB was positively correlated with depressive symptoms among women [48]. Another study with an adolescent sample, conducted in China demonstrated that girls were more likely than boys to have an overestimated body perception and higher levels of depression and anxiety [49].

Furthermore, the rapid growth and development of the adolescent body increases perceptual interest in the body and requires adolescents to revise their perception of body type, making precise recognition of the adolescent body one of the most important developmental tasks. Therefore, it is expected that an accurate body perception is essential for the healthy development of adolescents, and an effective plan for their dietary management and exercise can be established if OBP matches OBB. Accordingly, education and strategy development are needed to help teenagers accurately recognize the adolescent body.

This study has several limitations. First, since the study utilized a cross-sectional design, it was not possible to precisely capture the temporal and sequential relationships for body perception, quality of life, and stress perception. Second, due to the use of secondary data, there were limited opportunities for considering clinical health data that may have influenced perceived obesity, quality of life, and stress perception. Future longitudinal studies are needed to more precisely capture the time-related aspects of body and stress perception. Third, in this study, the correlation coefficient between well-being and stress perception was quite high at −.524 (*p* < .01). Therefore, in order to obtain a more accurate and in-depth assessment of mental health, it is necessary to examine additional aspects of mental health (e.g., self-esteem, depression), using multi-dimensional measures. Fourth, in this study, BMI was calculated using self-reported height and weight, which may be inaccurate. Therefore, it is necessary in future studies to calculate BMI through direct measurement of height and weight.

## Conclusion

OBB and OBP coincided in 51.5% of the sample. However, the degree of obesity was overestimated and underestimated by 29.4 and 19.0% of the sample, respectively. In addition, OBB and OBP were found to vary by gender, age, economic status, and academic achievement.

The main effect of OBB was not statistically significant, but the main effects of OBP and the interaction effect of OBP and gender on well-being and stress perception were all statistically significant. Well-being scores were higher for males than females, and the scores for the group with perceptual obesity were the lowest. There was no difference in well-being scores between females who perceived themselves as underweight and those who perceived themselves as having a healthy weight, but the scores of men who perceived themselves as underweight were higher than those of men who perceived themselves as having a healthy weight. For stress perception, females scored higher than males. Also, both males and females who perceived themselves as obese had the highest stress perception scores. The well-being and stress perception are influenced by obesity based on perception rather than obesity based on BMI, and this influence varies according to gender in adolescents.

## Data Availability

The datasets generated during and/or analyzed in the current study are available in the Korea Centers for Disease Control and Prevention repository, https://www.cdc.go.kr/yhs.

## References

[1] Koplan JP, Liverman CT, Kraak VA (2005). Preventing childhood obesity: health in the balance.

[2] Wang Y (2001). Cross-national comparison of childhood obesity: the epidemic and the relationship between obesity and socioeconomic status. Int J Epidemiol.

[3] Statistics Korea. 2018 Youth statistics. Daejeon: Statistics Korea; 2019.

[4] Daniels SR, Arnett DK, Eckel RH, Gidding SS, Hayman LL, Kumanyika S, Robinson TN, Scott BJ, Jeor S, Williams CL (2005). Overweight in children and adolescents: pathophysiology, consequences, prevention, and treatment. Circulation.

[5] Dehghan M, Akhtar-Danesh N, Merchant AT (2005). Childhood obesity, prevalence and prevention. Nutr J.

[6] Cancer Research UK. How being overweight causes cancer? https://www.cancerresearchuk.org/about-cancer/ causes-of-cancer/obesity-weight-and-cancer/does-obesity-cause-cancer. Accessed 10 Jun 2021.

[7] Bays HE, Bazata DD, Fox KM, Grandy S, Gavin JR, Group SS (2009). Perceived body image in men and women with type 2 diabetes mellitus: correlation of body mass index with the figure rating scale. Nutr J.

[8] Bozorgmanesh M, Sardarinia M, Hajsheikholeslami F, Azizi F, Hadaegh F (2016). CVD-predictive performances of "a body shape index" versus simple anthropometric measures: Tehran lipid and glucose study. Eur J Nutr.

[9] Goodman E, Whitasker RC (2002). A prospective study of the role of depression in the development and persistence of adolescent obesity. Pediatrics.

[10] Mond J, Mitchison D, Latner J, Hay P, Owen C, Rodgers B (2013). Quality of life impairment associated with body dissatisfaction in a general population sample of women. BMC Public Health.

[11] Paeratakul S, White MA, Williamson DA, Ryan DH, Bray GA (2002). Sex, race/ethnicity, socioeconomic status, and BMI in relation to self-perception of overweight. Obes Res.

[12] Schwartz MB, Brownell KD (2004). Obesity and body image. Body Image.

[13] Kim SW (2001). Body Wight and body image: a risk factor analysis in Korea. Korean Survey Research.

[14] Kim HA (2004). Comparison of normal weight vs obese children in terms of family factors, eating habits and socio-cognitive factors. J Korean Acad Child Health Nurs.

[15] Hwang IC, Lee KS, Park DK, Jung EY, Choi CH, Cho SJ, Bae SM (2001). Association with self-perception for obesity and mental health among Korean adolescent. J Korean Acad Child Adolesc Psychiatry.

[16] Jeon YS, Ahn HS (2006). Influence of subjective perception of body image and weight management on obesity stress in college women. J Korean Soc Food Sci Nutr Esthetic Cosmeceutics.

[17] Jeon SM. Attitude of body weight control, eating disorder and stress by degree of obesity of middle school students in Kunsan area. Master dissertation. Kunsan: Kunsan University. 2009.

[18] Nam SJ, Park JH (2013). Adolescents’ satisfaction of body and self-esteem according to obesity and subjective perception of body: verification of moderating effect of sex. Korean J Obes.

[19] Song MK, Ha JH, Park DH, Ryu SH, Oh JH, Yu JH (2010). Effect of body image and eating attitude on depressive mod and suicide ideation in female adolescents. Korean J Psychosom Med.

[20] Chin HJ, Chang KJ (2005). College students' attitude toward body weight control, health related lifestyle and dietary behavior by self-perception on body image and obesity index. J Korean Soc Food Sci Nutr.

[21] Won KH (2003). A comparative study of the degree satisfaction of body image and sex-role identify between primary school boys and girls. J Korean Soc School Health.

[22] Niu J, Seo DC, Lohrmann DK (2014). Weight perception and dietary intake among Chinese youth, 2004–2009. Int J Behav Med.

[23] Gaylis JB, Levy SS, Hong MY (2020). Relationships between body weight perception, body mass index, physical activity, and food choices in Southern California male and female adolescents. Int J Adolesc Youth.

[24] Solmi F, Sharpe H, Gage SH, Maddock J, Lewis G, Patalay P (2020). Changes in the prevalence and correlates of weight-control behaviors and weight perception in adolescents in the UK, 1986–2015. JAMA Pediatr.

[25] Oh DN, Kim EM, Kim S (2013). Weigh control behaviors and correlates in Korean adolescents. J Korea Contents Assoc.

[26] Hartman-Munick SM, Gordon AR, Guss C (2020). Adolescent body image: influencing factors and the clinician’s role. Curr Opin Pediatr.

[27] Pakpour AH, Chen CY, Lin CY, Strong C, Tsai MC, Lin YC (2019). The relationship between children’s overweight and quality of life: a comparison of sizing me up, PedsQL and KidKINDL. Int J Clin Health Psychol.

[28] Pakpour AH, Tsai MC, Lin YC, Strong C, Latner JD, Fung XC, Lin C-Y, Tsang HW (2019). Psychometric properties and measurement invariance of the weight self-stigma questionnaire and weight Bias internalization scale in children and adolescents. Int J Clin Health Psychol.

[29] Park B, Cho HN, Choi E, Seo DH, Kim S, Park YR, Choi HS, Rhee Y (2019). Self-perceptions of body weight status according to age-groups among Korean women: a nationwide population-based survey. PLoS One.

[30] Kim M, Kim S, Kim W, Choi HJ (2021). Mental health of people with distorted body weight perception using medicinal remedies: a representative study. Int J Clin Health Psychol.

[31] Chang VW, Christakis NA (2003). Self-perception of weight appropriateness in the United States. Am J Prev Med.

[32] CDC, 2015. About Child & Teen BMI | Healthy Weight. Centers for Disease Control and Prevention Retrieved Jun 10, 2021, from Center for Disease Control and Prevention website: http://www.cdc.gov/healthyweight/assessing/bmi/childrens_ bmi/about_childrens_bmi.html.

[33] Chu MA, Choe BH (2010). Obesity and metabolic syndrome among children and adolescents in Korea. J Korean Med Assoc.

[34] Korea Disease Control and Prevention Agency and The Korean Pediatric Society. 2017 Growth Charts for Children and Adolescents Commentary. Osong: Korea Disease Control and Prevention Agency; 2017.

[35] Ahmad F, Jhajj AK, Stewart DE (2014). Madeline Burghardt4 and Arlene S Bierman5, Single item measures of self-rated mental health: a scoping review. BMC Health Services Research.

[36] Cheung PC, Ip PL, Lam S, Bibby H (2007). A study on body weight perception and weight control behaviors among adolescents in Hong Kong. Hong Kong Med J.

[37] Pritchard ME, King SL, Czajka-Narins DM (1997). Adolescent body mass indices and self-perception. Adolescence.

[38] Meadow RM, Weis L (1992). Women's Conflicts about Eating and Sexuality: The Relationship between Food and Sex.

[39] Hayes D, Ross CE (1987). Concern with appearance, health belief, and eating habits. J Health Soc Behav.

[40] Kim OS (1998). Obesity and weight control behaviors of middle and high school girl students. Nurs Sc.

[41] Field A (2013). Discovering Statistics using IBM SPSS 4th edition.

[42] Rosenthal R, Rosnow RL, Winer BJ (1991). Essentials of behavioral research: Methods and data analysis.

[43] Brown DR, Michels KM (1991). Statistical Principles in experimental design.

[44] Kim Y, Austin SB, Subramanian SV, Kawachi I (2018). Body weight perception, disordered weight control behaviors, and depressive symptoms among Korean adults: The Korea National Health and Nutrition Examination Survey 2014. PLoS One.

[45] Solmi F, Sharpe H, Gage S, Maddock J, Lewis G, Patalay P (2021). Change in the prevalence and correlateds of weight-control behavior and weight perception in adolecents in the UK. 1986-2015. JAMA Pediatr.

[46] Kim H-S, Jang J-W Lee K-M, Jung S-P, Keum SH (2015). Association between actual or perceptional weight status and mental health issues in adolescents in Korea. Korean J Health Promot.

[47] Choi BY, Choi MO (2008). The survey for body shape recognition fatness knowledge and fatness stress of college woman. J Korean Soc Cosmetol.

[48] Abara W, Annang L, Spencer SM, Fairchild AJ, Billings D (2014). Understanding internet sex-seeking behaviour and sexual risk among young men who have sex with men: evidences from a cross-sectional study. Sex Transm Infect.

[49] Xie B, Chou CP, Spruijt-Metz D, Reynolds K, Palmer PH, Wu Q, Gallaher P, Johnson CA (2011). Longitudinal analysis of weight perception and psychological factors in Chinese adolescents. Am J Health Behav.

